# A Spatial Millimetre-Wave Radar Point Cloud Dataset for Health Applications

**DOI:** 10.1038/s41597-026-07808-w

**Published:** 2026-08-10

**Authors:** Kailu Guo, Elif Dogu, Khalid Z. Rajab

**Affiliations:** 1 School of Electronic Engineering and Computer Science, Queen Mary University of London, London, E1 4NS United Kingdom; 2 School of Immunology and Microbial Sciences, King’s College London, London, SE1 9RT United Kingdom

## Abstract

This work presents a controlled millimetre-wave radar point-cloud dataset for analysing guided daily and rehabilitation-related movements in standardised indoor settings. The dataset is designed to support radar-based human motion analysis: radar point clouds serve as the primary sensing modality, while the Azure Kinect provides 3D skeleton labels for supervised learning and validation. Data were collected from 26 healthy, able-bodied adults aged 25–57 years. Participants followed reference videos to perform a set of daily and rehabilitation-related movements under two visual conditions: normal lighting and low-light settings. After quality control, the release contains approximately 3.47 minutes of retained movement data per participant per lighting condition. The original recording-level data include 265,487 radar frames and 322,833 Kinect-derived skeleton-label frames, while the processed aligned data contain 137,717 paired radar–skeleton frames. Each aligned radar frame is annotated with an action label, subject ID, and corresponding Kinect-derived 3D skeleton. By combining radar point clouds with aligned skeleton labels across controlled lighting conditions, the dataset supports tasks such as radar-based action classification, identity recognition, and keypoint detection.

## Background & Summary

The healthcare domain has witnessed increasing interest in privacy-sensitive methods for human activity recognition (HAR) and pose estimation, particularly in the context of remote patient monitoring, personalised care, and home-based rehabilitation. Rehabilitative exercise can support physical autonomy, but access to guided therapy may be limited by clinical resources, travel distance, and personal constraints. Remote monitoring systems^1^ may assist rehabilitation studies by recording movement-related signals and supporting quantitative assessment outside conventional laboratory settings.

Wearable devices have been widely explored for motion monitoring^2^, but their use can be affected by adherence and comfort. Vision-based systems provide rich spatial information but may raise privacy concerns in personal spaces. Millimetre-wave (mm-wave) radar is a non-contact sensing modality that does not capture optical images and can operate under different lighting conditions^3^. It has been investigated for activity classification^4,5^, fall risk screening^6–8^, and fall detection^9^. These properties make radar a relevant sensing modality for research on home-based and rehabilitation-oriented motion analysis.

Table 1 summarises selected radar point-cloud datasets in terms of participant number, frame count, point attributes, skeleton annotations, action types, lighting conditions, and supported reuse tasks. Existing datasets differ in cohort size, participant demographics, data representation, annotation density, and acquisition conditions. Some datasets include a small number of participants^10–12^, while others mainly involve young adult participants^13,14^. The number of radar point attributes and annotated skeleton keypoints also varies across datasets, which affects the types of motion-analysis tasks that can be supported. In addition, many existing datasets focus on general daily activities, whereas rehabilitation-orientated studies often require movements involving reaching, balance control, posture transition, and limb coordination. All of these datasets provide only processed radar point clouds. In contrast, our dataset includes both original data and processed point clouds, and was collected under diverse lighting conditions.

**Table 1:** Comparison of selected radar point-cloud datasets and the dataset presented in this work.

Dataset	Subjects	Frame Size	Point Attributes	KP-Points	Motion Kinds	Light	Task
RadHAR^11,25^	2	167k	—	—	Daily	Light	Act
MARS^10,26^	4	40k	5	19	Daily	Light	KP
mRI^13,27^	20	160k	5	—	Daily	Light	KP
MiliPoint^12,28^	11	213k	3	9 / 18	Daily	Light	Act, Iden, KP
MM-Fi^14,29^	40	320k	3	17	Daily and Rehab	Light	Act, KP
This dataset	26	588k	8	32	Daily and Rehab	Light / Dark	Act, Iden, KP

Note: “Act” = Action Classification, “KP” = Keypoint Detection, “Iden” = Identification.

This dataset was developed as a controlled healthy-reference resource for radar-based motion analysis. Rather than targeting unconstrained clinical rehabilitation sessions, the objective is to provide a standardised baseline for early-stage algorithm development, facilitating controlled data variability while minimising participant risk.

The dataset includes both unprocessed original radar data and pre-processed radar point clouds. This structure enables researchers to either apply the existing pre-processed baseline or develop custom signal-processing pipelines for future applications. The pre-processed radar data incorporates a diverse set of point features, more than the other datasets. The dataset contains both daily activities and rehabilitation-related movements collected under both normal-light and low-light conditions. The rehabilitation-related movements were selected with input from physiotherapists and clinicians, covering guided movement patterns associated with reaching, balance control, posture transition, and limb coordination. The provided annotations include action labels, subject identities, and Azure Kinect-derived 3D skeleton labels. The skeleton data contains 32 keypoints, offering a higher joint density compared to existing radar-based human motion datasets.

Regarding reuse potential, these dataset characteristics directly support multiple downstream tasks, including action recognition, identity recognition, and human pose estimation.

## Methods

### Ethics statement

Participation was voluntary, and participants received compensation in accordance with UK regulations and the approved institutional protocol. Prior to data collection, all participants were informed about the study aims, experimental procedures, potential risks, and the planned public release of the dataset. The released data would be anonymised and would contain only motion-related radar point clouds, skeleton coordinates, action labels, and subject ID, without any directly identifiable personal information. Written informed consent was obtained from all participants for participation in the study and for sharing the anonymised motion-related data in a public repository. Participants were also informed before the experiment that they could contact the research team if they wished to request removal of their data from future public releases. All data were handled in accordance with institutional ethical guidelines and applicable data protection requirements. The study was approved by the Queen Mary Ethics of Research Committee (Reference No: QME24.0392).

### Experimental setup

**Fig. 1:**
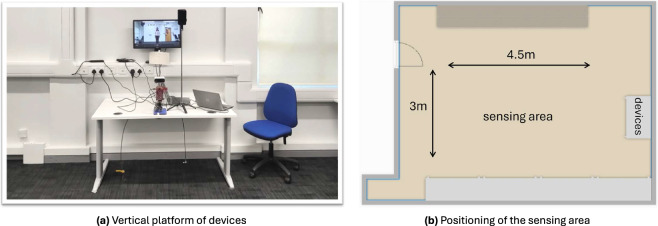
Data collection environment and setup.

The experimental setup consists of a vertical platform supporting both the mm-wave radar signal processing device and the Azure Kinect camera, a large screen positioned above the platform to display a guidance video for assisting participants with the required movements, and a laptop used to control and coordinate all components of the system (Fig. 1). The radar device was placed on a table with a height of 73 cm and securely fixed to ensure stability throughout the experiment. The Azure Kinect was mounted directly above the radar and used to provide reference skeleton annotations for subsequent data validation and analysis.

The experiments were conducted in a typical controlled indoor room with plain painted walls, a carpeted floor, and several ordinary tables with laminate board tops and grey powder-coated metal legs around the activity area. No large metallic objects, mirrors, glass walls, or other intentionally highly reflective surfaces were placed in this room. The main surrounding surfaces consisted of common indoor materials rather than strong specular reflectors.

The physical room was approximately 6.5 m × 7 m, whereas the effective capture area shown in Fig. 1(b) was approximately 4.5 m × 3 m and corresponded to the retained sensing region used for participant motion recording and radar point filtering. Radar points outside this predefined activity area were discarded, as they were more likely to originate from environmental reflections, multipath effects, or irrelevant background objects. Data collection was conducted under two lighting scenarios: a well-lit environment and a low-light environment. These two conditions were included to record participant movements under different visual conditions.

### Participant recruitment

26 healthy adults with no known mobility limitations were recruited for this study. Participant ages range from 25 to 57 years $(\mu \,=\,34.15,\sigma \,=\,\mathrm{7.45)}$, heights from 159 cm to 186 cm $(\mu \,=\,172.1\,cm,\sigma \,=\,7.00\,cm)$, weights from 52 kg to 97 kg $(\mu \,=\,71.4\,kg,\sigma \,=\,11.5\,kg)$, and body mass index (BMI) values from 17.99 to 30.11 $(\mu \,=\,24.11,\sigma \,=\,\mathrm{3.49)}$.

### Sensor modalities

#### Point cloud from mm-wave radar

A Texas Instruments IWR6843AOPEVM was employed, which is a Frequency-Modulated Continuous Wave (FMCW), multiple-input multiple-output (MIMO) radar system operating in the unlicensed 60 GHz frequency band. It was configured specifically for the detection and tracking of human movement^15,16^ using the TI 3D People Counting firmware^17^. Detection of points is performed using the constant false alarm rate (CFAR) algorithm applied to the radar data cube. For each detected point p, the output includes local three-dimensional coordinates $({X}_{p},{Y}_{p},{Z}_{p})$, and Doppler velocity $({D}_{p})$, all measured relative to the radar sensor. The radar's native spherical coordinate system $({\rho }_{i},{\varphi }_{i},{\theta }_{i})$ is transformed into Cartesian coordinates for further analysis and integration. Detailed configuration parameters of the radar system are summarised in Table 2.

**Table 2:** Radar configuration.

Parameter	Value	Unit	Parameter	Value	Unit
Start Frequency	60.75	GHz	Slope	54.71	MHz/*μ*s
Samples per chirp	96	—	Chirps per frame	144	—
Frame duration	55	ms	Sampling Rate	2.950	Msps
Bandwidth	1780	MHz	Range Resolution	84	mm
Velocity resolution	0.19	m/s	Number of RX antennas	4	—
Number of TX antennas	3	—			

### RGB-D frames from Azure Kinect

To provide a reference for radar-based motion analysis, we synchronously recorded skeleton information using Azure Kinect during data collection. Azure Kinect offers markerless RGB-D sensing through a time-of-flight depth camera and an RGB camera, and its Body Tracking SDK estimates 3D skeletons with 32 joints at 30 Hz for each tracked body^18^. It was selected as a practical reference modality because it is easier to deploy than multi-camera marker-based motion-capture systems, while previous studies have reported useful depth accuracy and body-tracking performance in controlled settings^19,20^. The Kinect-derived skeletons are used for spatial calibration, temporal alignment, data quality inspection, and supervised evaluation of radar-based models. They are not used as model input during radar-only inference.

### Data collection

**Fig. 2:**
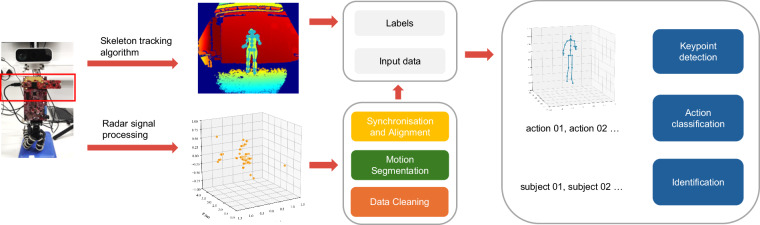
Overview of the data collecting system pipeline with three benchmark tasks.

Figure 2 illustrates the overall system pipeline. Each participant spent approximately 20 minutes completing the experimental procedure, including the study introduction, explanation of experimental requirements, consent confirmation, movement demonstration, and data recording.

During recording, participants stood at a fixed position approximately 3.5 m from the sensor platform to remain within the shared sensing region of the mm-wave radar and Azure Kinect. Participants were instructed to follow the reference video (https://youtu.be/WBGPFIWC_50) displayed on the monitor and reproduce the demonstrated movements as consistently as possible. The movement set contained 21 predefined categories, shown in Table 3. Each movement was repeated three times. After each movement, participants returned to the initial standing posture, with both arms naturally relaxed at the sides of the body, feet together, and the body kept still until the next movement began. The duration of each movement and the short pauses between consecutive movements are listed in Table 3.

**Table 3:** Daily and rehabilitation movement categories used in the guidance video.

ID	Movement	Duration	Pause	ID	Movement	Duration	Pause
**Daily movements**							
1	Walk on the spot	6 s	3 s	5	Throw to left	7 s	3 s
2	Wave left arm	5 s	1.5 s	6	Throw to right	7 s	3 s
3	Wave right arm	5 s	2 s	7	Twist to left	8 s	2 s
4	Pick up from ground	9 s	3 s	8	Twist to right	8 s	2 s
**Rehabilitation exercises**							
9	Chest expanding horizontal	4 s	3 s	16	Left side lunge	9 s	2 s
10	Left arm side reach	7 s	2 s	17	Right side lunge	9 s	1.5 s
11	Right arm side reach	7 s	2 s	18	Jump up	5 s	2 s
12	Left front lunge	9 s	2 s	19	Left side kick	6 s	2 s
13	Right front lunge	9 s	1.5 s	20	Right side kick	6 s	2 s
14	Squat	12 s	2 s	21	Chest expanding vertical	7 s	2 s
15	Sky reach and toe touch	9 s	2 s				

Each movement was repeated three times.

To ensure that the full movement sequence was captured, approximately 5 s of static standing was recorded before and after each group of actions. These additional static periods were used to facilitate segmentation and quality checking, and were removed as much as possible during data preprocessing. The full movement set was recorded under both normal-light and low-light conditions.

### Data processing

#### Calibration

**Fig. 3:**
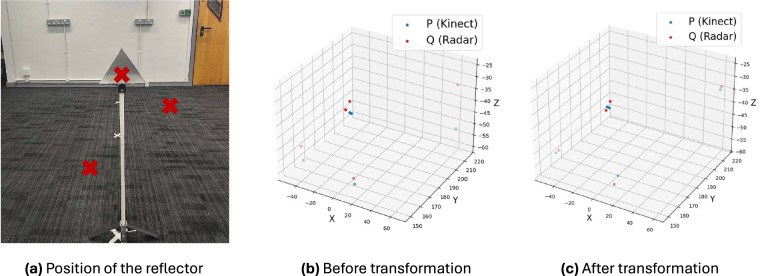
Calibration of Kinect and radar coordinates.

The radar operates within a coordinate system centred at its transmitter, whereas the Azure Kinect defines its coordinate system with the optical centre of the depth camera as the origin. To enhance the accuracy of downstream tasks, it is essential to align the coordinate frames by transforming the keypoint positions from the camera coordinate system to that of the radar. For this purpose, a corner reflector is positioned at multiple known locations to serve as an anchor point (Fig. 3).

For radar, signals are predominantly reflected when the reflector's centre is aligned with the radar's line of sight. In the case of the Azure Kinect, which integrates both depth and RGB cameras, object recognition techniques are employed to detect the triangular reflector. Subsequently, the centre point's position is calculated within the Kinect coordinate system.

Table 4 enumerates the selected calibration points. A real point is concurrently observed by both the radar device and the Azure Kinect. These devices independently compute the point's position within their respective coordinate systems. The transformation is performed using Singular Value Decomposition (SVD) as part of the standard Procrustes alignment method, which involves the following steps:

1. **Centroid Alignment:** We compute the centroids of both point sets and subtract them from the original points to obtain centred sets ${{\bf{P}}}_{c}$ and ${{\bf{Q}}}_{c}$.
2. **Singular Value Decomposition (SVD):** We calculate the covariance matrix ${\bf{H}}={{\bf{P}}}_{c}^{\top }{{\bf{Q}}}_{c}$ and perform SVD: ${\bf{H}}={\bf{U}}{\boldsymbol{\Sigma }}{{\bf{V}}}^{\top }$. The optimal rotation is then given by ${\bf{R}}={\bf{V}}{{\bf{U}}}^{\top }$.
3. **Reflection Correction:** If ${\rm{d}}{\rm{e}}{\rm{t}}({\bf{R}}\mathrm{) < 0}$, we correct for improper rotation by flipping the sign of the last row of ${{\bf{V}}}^{\top }$ and recomputing ${\bf{R}}$.
4. **Translation Vector:** The translation vector is computed as ${\bf{t}}=\bar{{\bf{Q}}}-{\bf{R}}\bar{{\bf{P}}}$.
5. **Transformation and Error Evaluation:** The original Kinect points are transformed using ${{\bf{P}}}_{{\rm{aligned}}}={\bf{R}}{\bf{P}}+{\bf{t}}$.

**Table 4:** Kinect vs radar anchor position comparison.

Kinect			Radar		
x(cm)	y(cm)	z(cm)	x(cm)	y(cm)	z(cm)
7.24	155.58	30.37	9.64	149.53	−26.89
9.32	155.34	30.45	8.85	154.18	−25.22
66.64	208.05	48.83	60.34	215.43	−33.45
10.23	156.67	60.19	8.99	157.05	−58.05
-50.9	161.44	59.40	−50.97	159.83	−52.73

Figure 3 shows the calibration points in the camera and radar coordinate systems before and after alignment. The resulting transformation parameters $({\bf{R}},{\bf{t}})$ are saved and subsequently used to align Kinect-derived skeleton labels with radar data. After applying the computed transformation, the Kinect points align closely with the corresponding radar points, with a mean Euclidean residual of 39.0 mm, an RMSE of 44.0 mm, and a maximum residual of 75.5 mm. The mean absolute residuals along the x/y/z axes were 10.9/33.1/12.3 mm.

#### Synchronisation

The radar device operates at 18.2 Hz, while the Azure Kinect captures at 30 Hz, leading to a natural temporal misalignment between their data streams. In our setup, both sensors were controlled by a single host computer, which triggered data capture and recorded a system timestamp immediately after each frame. This shared system clock ensured consistent timestamp formatting and comparability across devices. To achieve frame-level alignment, a nearest-neighbour timestamp matching method was applied. The residual temporal mismatch after this software-based synchronisation was quantified using the absolute timestamp difference between matched radar and Kinect frames, and the empirical synchronisation-error statistics are reported in Table 5.

**Table 5:** Quantitative analysis of timestamp synchronisation errors after nearest-timestamp alignment.

Pipeline Scenario	Mean (ms)	Median (ms)	RMSE (ms)	p95 (ms)	p99 (ms)	p99.9 (ms)	Max (ms)
Full Deployed Pipeline	8.52	8.42	12.19	16.99	22.14	30.86	491.25
Filtered Pipeline	8.41	8.41	10.64	16.98	22.03	30.52	77.32

As shown in Table 5, the deployed timestamp-based pipeline achieved a mean error of 8.52 ms, a median error of 8.42 ms, and an RMSE of 12.19 ms, with p95, p99, and p99.9 errors of 16.99 ms, 22.14 ms, and 30.86 ms. Isolated timestamp interruptions affected 61 out of 137,717 frames and produced a maximum error of 491.25 ms; after excluding the affected recordings, the maximum error decreased to 77.32 ms while the mean and median remained at 8.41 ms. Overall, the procedure provides stable synchronisation for the current controlled acquisition protocol, although residual timing errors may still affect fast or abrupt movements. Hardware triggering was not used because the Azure Kinect wired synchronisation mode is designed for multi-Kinect setups, and the radar pipeline did not provide a directly compatible shared trigger or common clock interface.

#### Motion segmentation

Each recording group contains approximately four consecutive actions separated by short static pauses. Motion segmentation was performed to extract the individual action intervals from each group. We used frame-wise Motion Energy (ME) computed from the Kinect-derived skeleton sequence as the primary segmentation cue, because the skeleton trajectories provide a compact representation of whole-body movement.

Before ME calculation, each joint coordinate trajectory was smoothed using a one-dimensional Gaussian filter to reduce frame-level jitter. For a skeleton sequence with *T* frames and *J* joints, let ${{\bf{p}}}_{t}^{(j)}\in {{\mathbb{R}}}^{3}$ denote the 3D coordinate of joint *j* at frame *t*. The ME at frame *t* is defined as

1 $${{\rm{ME}}}_{t}=\mathop{\sum }\limits_{j\mathrm{=1}}^{J}{\Vert {{\bf{p}}}_{t}^{(j)}-{{\bf{p}}}_{t-1}^{(j)}\Vert }_{2},\,t=\mathrm{2,}\ldots ,T,$$

with ME_1_ = 0 to preserve the original sequence length.

The segmentation threshold was determined dynamically for each recorded group rather than fixed across subjects or actions. Let ${\rm{ME}}=\{{{\rm{ME}}}_{1},{{\rm{ME}}}_{2},\ldots ,{{\rm{ME}}}_{T}\}$ denote the motion-energy sequence of a recorded group. We applied a sliding window of length *L* = 60 to this sequence, where the *i*-th window is defined as

$${W}_{i}=\{{{\rm{ME}}}_{i},{{\rm{ME}}}_{i+1},\ldots ,{{\rm{ME}}}_{i+L-1}\mathrm{\}}.$$

For each window *W*_*i*_, we computed the local motion variation as

2 $${D}_{i}=\mathop{{\rm{m}}{\rm{a}}{\rm{x}}}\limits_{t\in \{i,\ldots ,i+L-\mathrm{1\}}}{{\rm{ME}}}_{t}-\mathop{{\rm{m}}{\rm{i}}{\rm{n}}}\limits_{t\in \{i,\ldots ,i+L-\mathrm{1\}}}{{\rm{ME}}}_{t}.$$

If ${D}_{i} < \delta$, the window was used as a candidate low-motion window for estimating the adaptive threshold. In our implementation, $\delta \,=\,100$, which was selected empirically to distinguish stable pauses from intervals with substantial movement variation. Table 6 present the number of valid frames and points retained for each action type after all processing steps.

**Table 6:** Movement categories and per-action statistics after segmentation. Values (Mean, Median, Min-Max) represent the total points per frame.

ID	Movement	Segments	Total frames	Mean	Median	Min–Max	Total Radar points
1	Walk on the spot	52	3,777	72.6	73.0	27–101	401,618
2	Wave left arm	52	4,306	82.8	81.0	48–124	242,502
3	Wave right arm	52	4,320	83.1	80.0	30–121	219,064
4	Pick up from ground	52	7,766	149.3	148.0	117–179	878,331
5	Throw to left	52	6,822	131.2	129.0	88–171	769,636
6	Throw to right	52	6,723	129.3	126.0	94–162	735,993
7	Twist to left	52	6,081	116.9	116.5	83–153	613,894
8	Twist to right	52	6,641	127.7	123.0	75–250	655,038
9	Chest expanding horizontal	52	4,510	86.7	82.5	66–159	384,771
10	Left arm side reach	52	6,673	128.3	125.0	96–163	627,147
11	Right arm side reach	52	6,870	132.1	131.0	106–174	633,542
12	Left front lunge	52	8,090	155.6	153.5	122–189	941,367
13	Right front lunge	52	8,347	160.5	158.5	128–223	972,451
14	Squat	52	8,706	167.4	168.0	132–214	848,276
15	Sky reach and toe touch	52	9,472	182.2	180.5	139–214	1,048,950
16	Left side lunge	52	8,119	156.1	155.0	116–188	773,329
17	Right side lunge	52	7,572	145.6	145.0	109–173	818,286
18	Jump up	52	5,117	98.4	101.5	51–115	533,247
19	Left side kick	52	5,578	107.3	107.0	66–133	496,361
20	Right side kick	52	6,446	124.0	124.0	105–146	591,656
21	Chest expanding vertical	52	5,781	111.2	108.0	83–155	408,757

Let ${{\mathscr{W}}}_{s}$ denote the set of candidate low-motion windows. The adaptive threshold *T*_ME_ was then computed as

3 $${T}_{{\rm{ME}}}=(\begin{array}{cc}{\rm{m}}{\rm{a}}{\rm{x}}\{{{\rm{ME}}}_{t}|{{\rm{ME}}}_{t}\in {W}_{i},\,{W}_{i}\in {{\mathscr{W}}}_{s}\}, & {\rm{if}}\,{{\mathscr{W}}}_{s}\ne \varnothing ,\\ {P}_{90}({\rm{ME}}), & {\rm{otherwise}},\end{array}$$

where ${P}_{90}({\rm{ME}})$ denotes the 90th percentile of the full ME sequence. When candidate low-motion windows are available, ${T}_{{\rm{ME}}}$ is the largest ME value observed within these windows. If no candidate low-motion window is detected, the 90th percentile of the full ME sequence is used as a fallback threshold.

Frames with ${{\rm{ME}}}_{t} < {T}_{{\rm{ME}}}$ were marked as stationary, and consecutive stationary frames were grouped into stationary intervals. In our implementation, a stationary interval was used as an action boundary only if it lasted for at least $K\,=\,60$ frames, corresponding to approximately 2 s for Kinect recordings at 30 Hz. This minimum-length criterion was used to avoid splitting actions at short pauses or transient fluctuations within a movement. Long stationary intervals were then used to separate consecutive action segments within each recorded group. This adaptive strategy accounts for differences in participant movement amplitude, execution speed, and skeleton tracking noise. All automatically detected action boundaries were visually inspected and adjusted where necessary before constructing the final aligned action segments.

After the above acquisition, alignment, segmentation, and quality-checking procedures, the final released dataset was constructed from the retained valid action segments.

## Data Records

**Fig. 4:**
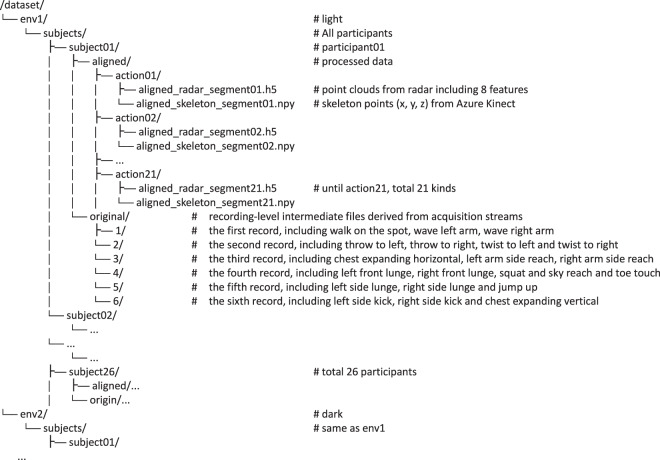
Overview of the proposed dataset structure.

The dataset^21^ is publicly available via Harvard Dataverse at 10.7910/DVN/UJBAPX. It contains recordings from 26 participants, identified as subject01 to subject26, collected under two environmental conditions denoted as env1 and env2. The dataset's structure is shown in Fig. 4.

**Fig. 5:**
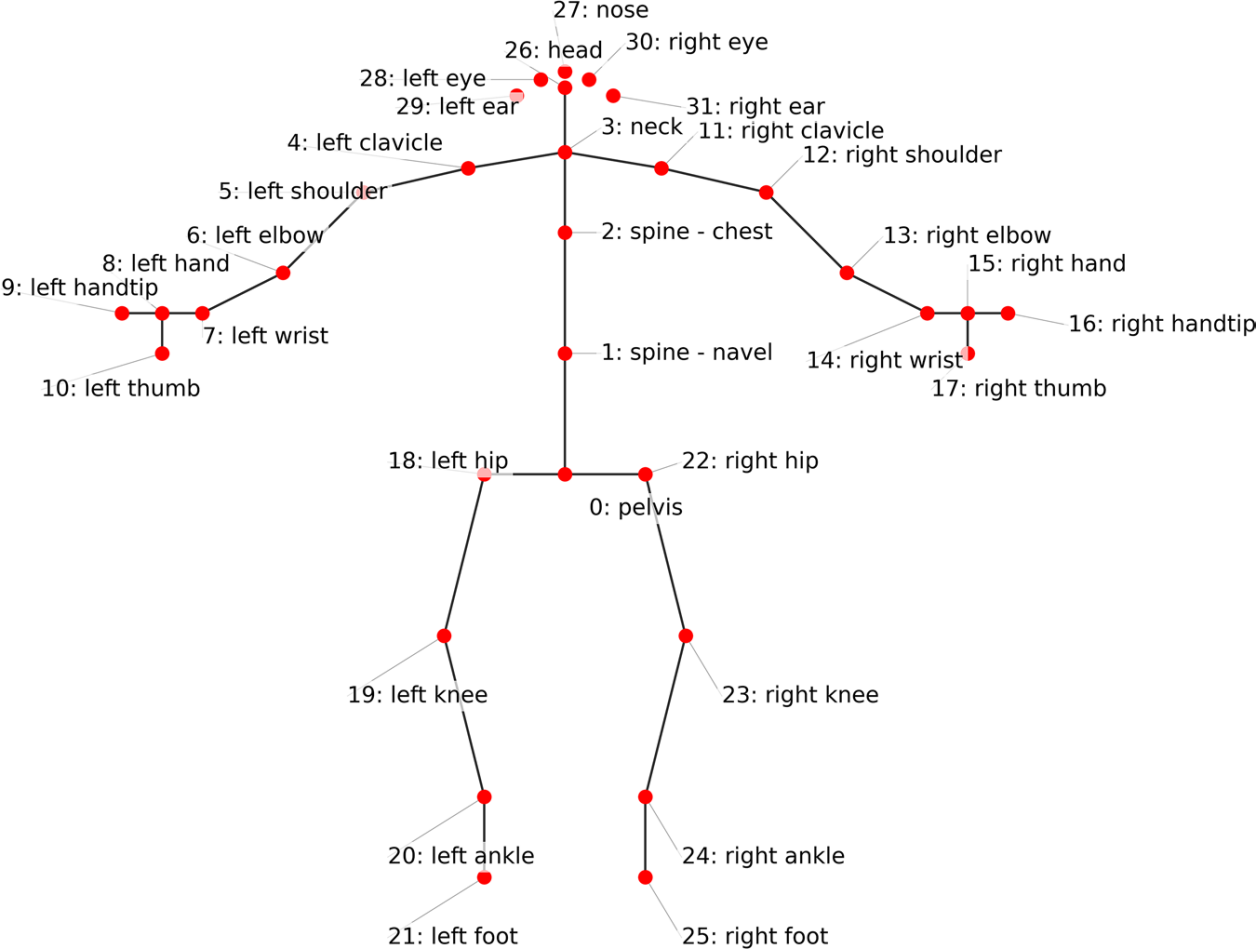
Visualisation of the 32 skeletal joints defined by Azure Kinect.

**Fig. 6:**
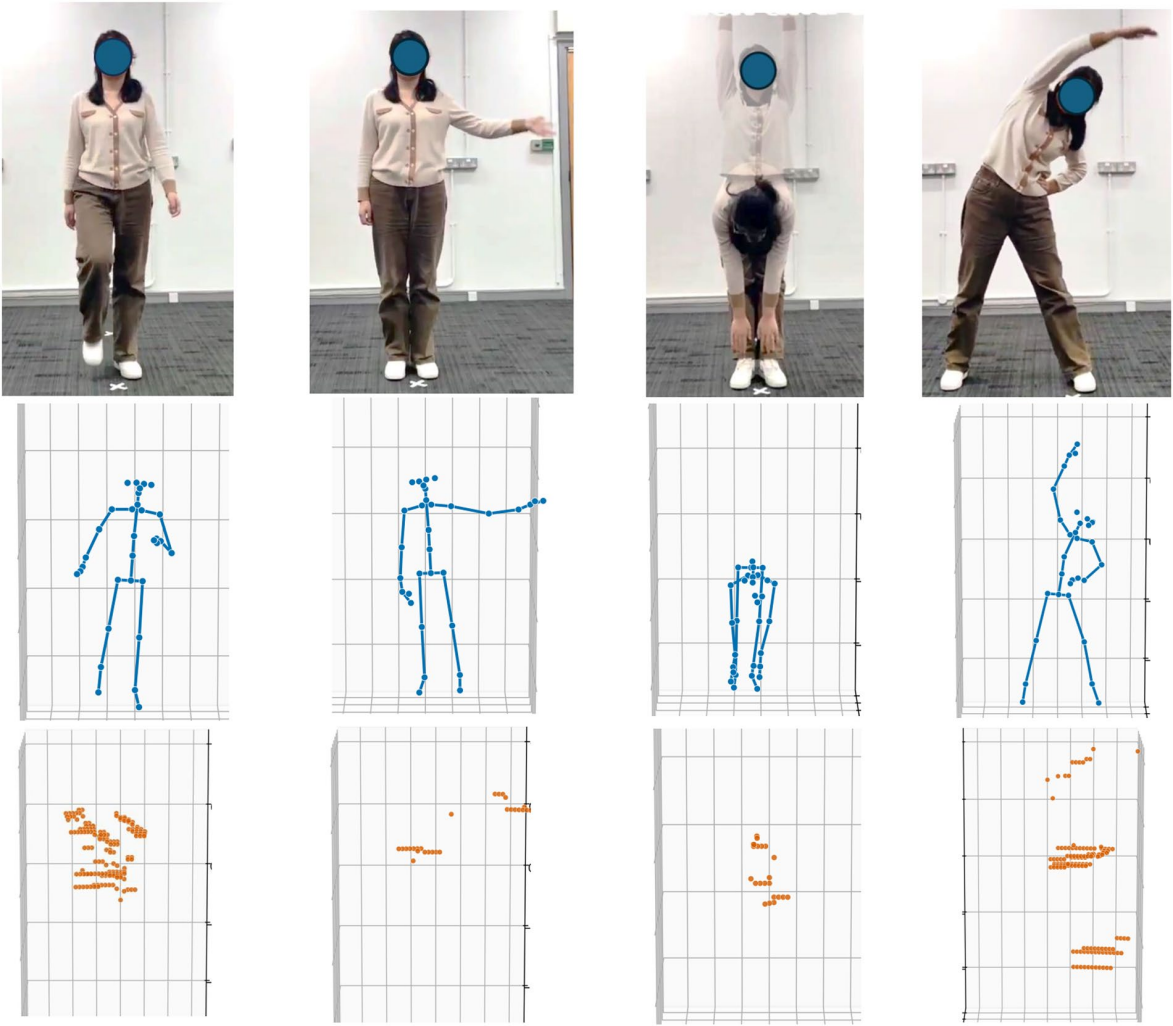
Examples of motion categories and their corresponding data representations. The identifiable images depict one of the authors, who consented to their publication. From left to right: (1) walk on the spot; (2) wave left arm; (3) sky reach and toe touch; and (4) right arm side reach.

Each aligned action segment is stored as an independent sample, with a .h5 file and a .npy file. Each.h5 file contains the radar point-cloud sequence and.npy file records the corresponding Kinect-derived skeleton labels. The radar point-cloud records include elevation, z_height, azimuth, doppler, range, x, y, snr. Radar spatial measurements are expressed in metres. The .npy file provides 3D skeletons with 32 joints (as shown in Fig. 5), stored as arrays of shape (*F*,32,3), where *F* is the number of frames. Figure 6 illustrates representative examples across the camera, Kinect-derived skeleton, and radar point-cloud modalities.

The release also includes recording-level intermediate files in the *original* folders. These files are derived from the original acquisition streams and are provided to support traceability and reuse. Each of the 312 raw recording groups contains derived Kinect files, including body_skeleton.npy, color_skeleton.npy, depth_skeleton.npy, depth_converted_skeleton.npy, timestamps.npy, and action_segments.txt. The timestamps.npy files store Unix timestamps in seconds, and the action_segments.txt files record the segmented action intervals used to construct the aligned samples. The sensor-native .mkv files are not included.

The radar files in the recording-level folders, such as.mat files, contain radar point cloud data exported from the acquisition pipeline for reuse, rather than the lowest-level device-native radar stream. Specifically, each file stores the point-cloud sequence over the entire recording without temporal alignment or frame-wise segmentation, where each frame consists of radar points represented by per-point features such as N, el, Z, az, Doppler, rng, X, Y, snr, size, and tid.

The released dataset also includes a README file describing the action dictionary, folder structure, file types, loading procedure, and field definitions.

## Technical Validation

### Dataset Completeness Check

We verified the completeness and structural consistency of the released dataset. As summarised in Table 7, all 1,092 aligned action directories contain complete radar–skeleton data pairs, with no missing or extra .h5/.npy files. The radar and skeleton streams also have matching frame counts for all aligned samples, resulting in 137,717 aligned frame pairs. No non-finite values, such as NaN or Inf, were detected in the released aligned files. Table 7 also summarises point-cloud quality statistics. Across the aligned frames, the radar data contain an average of 98.7 points per frame, with a median of 109 points per frame. The global empty-frame rate is 1.35%.

**Table 7:** Data integrity checks and frame-level synchronisation consistency.

Integrity Check Item	Result	Point Cloud Quality	Value
Aligned Action Directories	1092 / 1092	Mean Points per Frame	98.7
Complete Radar/Skeleton Pairs	1092 / 1092	Median Points per Frame	109
Non-finite (NaN/Inf) Values	0	Empty Radar Frames	1.35%
Total Aligned Radar Frames	137,717	Total Skeleton Frames	137,717

### Motion-signal consistency

**Fig. 7:**
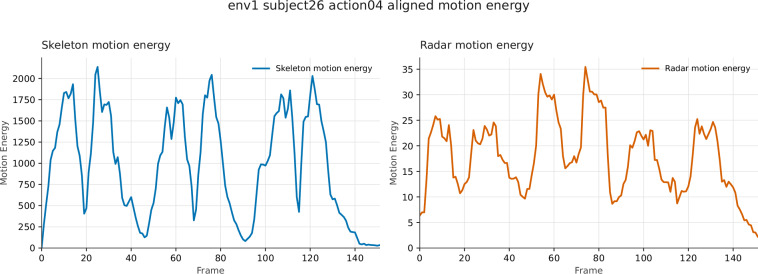
Motion-energy comparison between temporally aligned Kinect-derived skeleton sequences and radar point-cloud sequences after timestamp-based synchronisation.

Motion energy was used as a qualitative check of whether the aligned Kinect and radar recordings captured motion changes during the same guided action sequence. As shown in Fig. 7, the Kinect-derived skeleton energy varies clearly across repeated movement phases, with lower-energy intervals corresponding to pauses or returns to the initial standing posture. The radar motion-energy trace also changes over the same segment, with several higher-energy periods broadly matching active movement phases.

### Radar–skeleton spatial consistency

We evaluated the spatial consistency between the calibrated radar point clouds and the Kinect-derived reference skeletons using point-to-skeleton distances.

For each radar point, we computed its 3D Euclidean distance to the nearest skeleton bone segment. A radar point was counted as a *human-ROI point* if this distance was no greater than 0.60 m, indicating that the radar return was located near the human body region. A stricter threshold of 0.35 m was used to define a *body-core point*, indicating that the radar return was closer to the skeleton structure. At the frame level, we considered a frame to have a *usable body return* if it contained at least 5 radar points within the human ROI. A frame was further considered to have a *good body return* if it satisfied all three conditions: at least 5 body-core points, and a body-core-to-ROI point ratio of at least 35%. These criteria are intended to assess whether radar points are generally present near the body, rather than requiring exact point-to-joint overlap.

Table 8 shows that most non-empty radar frames contain returns near the Kinect-derived skeleton. Specifically, 86.64% of non-empty frames contain at least one radar point within the human ROI, and 83.97% contain usable body returns. A smaller proportion, 60.70%, satisfies the stricter good-body-return criterion, which additionally requires sufficient body-core points and a minimum body-core-to-ROI ratio. At the point level, each non-empty radar frame contains 100.06 radar points on average, of which 76.71 fall within the human ROI and 38.23 fall within the body-core region. This indicates that radar returns are commonly present around the body, but only part of the point cloud lies close to the skeleton structure. This pattern is consistent with the sparse and reflection-dependent nature of mm-wave radar point clouds, where returns may come from body surfaces, moving limbs, clothing, and surrounding reflections rather than directly from anatomical joint locations.

**Table 8:** Radar–skeleton spatial consistency based on point-to-skeleton distance. Percentages are computed over non-empty radar frames.

Metric	Value	Percentage
Frames with radar points in human ROI	117,707	86.64%
Frames with usable body returns	114,080	83.97%
Frames with good body returns	82,465	60.70%
Mean radar points per non-empty frame	100.06	—
Mean human-ROI points per non-empty frame	76.71	—
Mean body-core points per non-empty frame	38.23	—

**Fig. 8:**
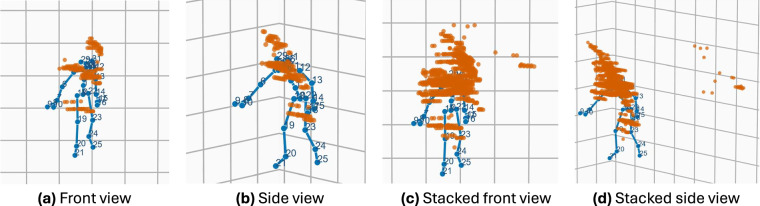
Overlay visualisations of calibrated radar point clouds and the corresponding Kinect-derived skeleton. (**a,b**) show single-frame radar point clouds from front and side views. (**c,d**) show point clouds after stacking three neighbouring radar frames.

Figure 8 qualitatively shows how the calibrated radar points are distributed around the Kinect-derived skeleton during a bending action. In the single-frame views, the radar point cloud is sparse and mainly concentrated around moving body parts, rather than uniformly covering the whole body. After stacking three neighbouring radar frames, the point cloud becomes noticeably denser and covers a larger body region, especially most of the upper body.

### Kinect skeleton label consistency

We assessed the temporal continuity of the Kinect-derived skeleton labels to verify their suitability. There were rare events in which a joint would displace by a non-physical distance, which we have identified as 'flickering'. For each action sequence, we measured the frame-to-frame displacement of all joints and flagged a transition as flickering if any joint moved by more than 1 m between two adjacent frames.

As shown in Table 9, among 1,092 action sequences there were 136,625 consecutive-frame transitions. Only 195 transitions were flagged as flickering, accounting for 0.143% of all transitions. This indicates that the Kinect-derived skeleton labels are generally temporally stable, although occasional flickering remains.

**Table 9:** Skeleton temporal continuity quality based on frame-to-frame joint displacement.

Metric	Value	Percentage
Frame transitions	136,625	—
Transitions with any joint displacement > 1 m	195	0.143%
Normal transitions	136,430	99.857%

**Fig. 9:**
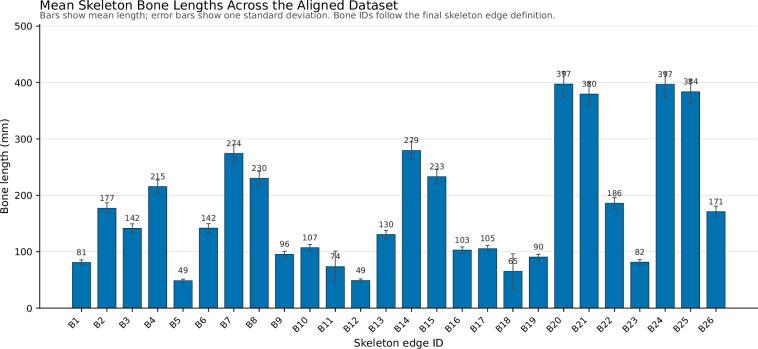
Bone-length statistics of the Azure Kinect skeleton annotations. Mean bone lengths are reported for the 26 predefined skeleton connections.

Figure 9 further shows that the mean bone lengths are within reasonable ranges. Bone length is computed for each of the 26 predefined Azure Kinect skeleton connections, including the head–neck link, pelvis–spine links, clavicle–arm–hand chains on both sides, and hip–knee–ankle–foot chains on both sides.

### Downstream data-usability checks

As an additional usability check, we evaluated representative point-cloud models on three downstream tasks: action classification, identity recognition, and keypoint detection. These experiments verify that the released radar point clouds, action labels, subject IDs, and Kinect-derived skeleton labels can be loaded and used in standard supervised learning pipelines. We evaluated three representative point cloud models: DGCNN^22^, PointNet++^23^, and Point Transformer^24^. The dataset was randomly split into training, validation, and test sets with a ratio of 8:1:1.

### Performance of benchmark tasks

We report the performance of three representative point-based models–DGCNN, Point Transformer, and PointNet++ in Table 10. For the action and identity classification tasks, Top-1 and Top-3 accuracies are reported, while mean localisation error (MLE) in centimetres is used for keypoint detection. For action classification, we employ a stack size of 50 frames and set *max_point* to 2048. In the identity recognition and keypoint detection tasks, the stack size is reduced to 16, with *max_point* limited to 1024 and 512.

**Table 10:** Performance comparison for identity recognition, action classification, and keypoint detection tasks.

Model	KP (MLE in cm ↓)	Act (Acc% ↑)		Iden (Acc% ↑)	
	32 points	Top1	Top3	Top1	Top3
DGCNN	23.66 ± 1.10	88.56 ± 0.23	89.77 ± 0.33	60.28 ± 1.78	85.31 ± 0.95
PtTrans	17.02 ± 0.93	89.89 ± 0.12	91.43 ± 0.17	85.62 ± 0.38	92.51 ± 0.23
PointNet++	17.74 ± 0.55	89.73 ± 0.08	90.42 ± 0.21	86.35 ± 0.43	92.35 ± 0.12

Note: PtTrans means Point Transformer

The three baseline models obtained Top-1 accuracy above 88% and Top-3 accuracy above 89% for action classification, indicating that the released radar point clouds and labels can support this benchmark task. Among them, Point Transformer achieves the best performance at 89.89% Top-1 and 91.43% Top-3, slightly outperforming PointNet++ and DGCNN. This indicates that the action patterns in the dataset are distinct and consistent enough to be recognised accurately by different models.

In contrast, identity classification is notably more challenging. The best Top-1 accuracy is 86.35% by PointNet++, while DGCNN lags behind at 60.28%. Identity recognition depends on subtler subject-specific cues such as body scale, limb proportion, reflection distribution, and individual motion style. The standardised action protocol may reduce inter-subject behavioural variation, making these cues less distinctive. This is particularly challenging for DGCNN, whose dynamic k-nearest-neighbour graph construction and local EdgeConv operations can be sensitive to sparse, noisy radar point clouds and multipath-induced neighbourhood changes. In comparison, PointNet++ and Point Transformer may better preserve identity-related information through hierarchical local-to-global aggregation and attention-based long-range modelling.

For keypoint detection, the evaluated models achieved MLE values comparable to those reported in radar-based benchmarks. Point Transformer obtained the lowest error of 17 cm, followed by PointNet++ (17.74 cm) and DGCNN (23.66 cm). These results should be interpreted in light of the task difficulty: each frame requires estimating 32 skeleton keypoints from sparse and noisy radar point clouds, where radar provides indirect reflections rather than dense body-surface observations. Some joints, especially distal ones, may have weak or unstable returns due to limited resolution, multipath effects, and variations in return strength. Although neighbouring frames were stacked to provide motion cues, each validation sample was treated independently rather than as part of a full temporal sequence, limiting the baselines' ability to exploit sequence-level continuity. Residual uncertainty from Kinect-derived reference keypoints and cross-modal calibration may also contribute to the measured localisation error.

### Light-condition comparison

The low-light condition was included to record participant movements under different visual conditions, rather than to test whether the sensors require visible illumination. In home-based or rehabilitation-related scenarios, reduced lighting may affect how participants perceive the surrounding space and perform guided movements. We therefore performed separate within-condition evaluations for normal-light and low-light recordings: models were trained and tested using only data from the same visual condition, following the same task protocol and metrics. The results in Table 11 are used as a data-usability check across the two visual conditions, rather than as a cross-condition generalisation test or evidence of clinical deployment robustness.

**Table 11:** Point Transformer performance under normal-light and low-light recordings.

Condition	KP (MLE in cm ↓)	Act (Acc% ↑)		Iden (Acc% ↑)	
	32 points	Top-1	Top-3	Top-1	Top-3
Normal light	16.29	90.34	92.39	66.28	86.94
Low light	16.55	90.82	93.26	66.78	86.67

For each condition, Point Transformer was trained and tested using data from the same lighting condition.

The results are comparable across the two conditions. For keypoint localisation, the MLE changes slightly from 16.29 cm under normal light to 16.55 cm under low light. Action recognition shows similar Top-1 / Top-3 accuracies, changing from 90.34% / 92.39% to 90.82% / 93.26%. Subject identification also remains close, with 66.28% / 86.94% under normal light and 66.78% / 86.67% under low light.

These results indicate that the released data from both visual conditions can be used in the same downstream supervised-learning pipeline and produce comparable within-condition baseline performance. Therefore, the normal-light and low-light recordings should be interpreted as two acquisition conditions covering visual-environment variation in real-world home-based or rehabilitation-related scenarios, with demonstrated downstream usability under the reported evaluation protocol.

## Usage Notes

We provide a code repository to help users read and work with the released dataset. The repository includes scripts for loading data, together with setup instructions and example usage. Users are encouraged to inspect the released samples using the provided visualisation code before model training or evaluation. The folder vis, included in the process_data of the code repository, can be used to visualise radar point clouds together with the corresponding Kinect-derived skeleton references.

The participant cohort should be considered when interpreting and reusing the dataset. This release includes healthy adults aged 25–57 years and is intended as a healthy-reference benchmark for radar-based motion analysis under standardised experimental conditions. In particular, the dataset is designed to help establish the boundaries of what may be considered "healthy" movement patterns from a data-driven perspective, providing a reference against which future datasets from clinical or mobility-impaired populations can be compared. It does not include older adults or individuals with balance impairments, neurological conditions, mobility limitations, or movement disorders. Models trained solely on this dataset should therefore not be assumed to generalise directly to clinical or mobility-impaired populations. Additional data from such cohorts would be needed to study pathological or compensatory motion patterns, or to evaluate contrastive learning, transfer learning, and domain adaptation between healthy-reference and clinical populations.

The skeleton annotations provided with the dataset should be regarded as Kinect-derived reference labels rather than ground-truth body joint coordinates. Although Azure Kinect provides a practical markerless reference modality for controlled radar data collection, its body-tracking output may not accurately reflect the true 3D skeleton coordinates and can be affected by tracking noise, temporal flickering, occlusion, fast motion, and sensor-specific artefacts. This limitation should be considered when using the labels for pose estimation, error analysis, or cross-modal evaluation.

The action set should be interpreted within the dataset's intended scope. It includes daily and rehabilitation-related movements selected with input from physiotherapists and clinicians, providing standardised recordings of rehabilitation-relevant movements performed by healthy participants. However, it is not a complete clinical rehabilitation dataset and does not capture spontaneous movement strategies, compensatory patterns, therapist-patient interactions, fatigue effects, or patient-specific adaptations. These limitations should be considered when reusing the dataset for rehabilitation-oriented motion analysis.

All actions were collected at a fixed position approximately 3.5 m from the sensor platform. This fixed-distance setting helps maintain a shared sensing region between the mm-wave radar and Azure Kinect, but limits spatial diversity. The current release does not capture variable-distance interactions, natural walking trajectories, turns, transfers, or activities performed across larger room spaces. It may therefore not directly represent larger or more cluttered hospital or rehabilitation rooms, where patients may move outside the optimal sensing region or be partially occluded by furniture, equipment, or caregivers.

The dataset and baseline experiments should be interpreted as a controlled foundation for radar-based motion analysis, rather than as evidence of clinical deployment performance. If radar point clouds can be used to estimate human motion reliably, they may support downstream applications such as behaviour analysis, abnormal movement detection, and remote reporting in future healthcare or rehabilitation systems. However, additional validation in patient cohorts and more natural clinical environments would be required before drawing conclusions about clinical use.

## Data Availability

The source code is available on GitHub at https://github.com/gklqmul/Radar3DforRehab, with release tag v1.0.0 and commit hash 3f2d373627da17d7153e1af021f2f45ee4ca7d58. All models in this study were implemented in PyTorch and trained from scratch using the released dataset, without relying on any external pre-trained weights. The experiments were conducted in an environment configured with CUDA 11.8. Training was performed on three high-performance computing servers, each equipped with six NVIDIA A100 GPUs and 512 GB of RAM. Across all experiments, training required more than 300 GPU hours in total, with six GPUs used in parallel. On average, each model required approximately seven hours to train across the three tasks. The Adam optimiser was adopted with a cosine annealing learning-rate schedule and an initial learning rate of 1×10^−5^. The batch size was set to 32, and a fixed random seed of 42 was used for reproducibility. Since the number of radar points varies across frames, zero padding and random downsampling were applied to standardise the input size. For convenience, the best-performing model weights are made available via Harvard Dataverse at 10.7910/DVN/YKS954.
